# Computer hardware for radiologists: Part I

**DOI:** 10.4103/0971-3026.69346

**Published:** 2010-08

**Authors:** IK Indrajit, A Alam

**Affiliations:** Department of Radiodiagnosis and Imaging, Command Hospital (Air Force), Bangalore - 560 007, Karnataka, India

**Keywords:** Chipset, computers, console, CPU, CT, hardware, motherboard, MRI, RAM, workstations

## Abstract

Computers are an integral part of modern radiology practice. They are used in different radiology modalities to acquire, process, and postprocess imaging data. They have had a dramatic influence on contemporary radiology practice. Their impact has extended further with the emergence of Digital Imaging and Communications in Medicine (DICOM), Picture Archiving and Communication System (PACS), Radiology information system (RIS) technology, and Teleradiology. A basic overview of computer hardware relevant to radiology practice is presented here. The key hardware components in a computer are the motherboard, central processor unit (CPU), the chipset, the random access memory (RAM), the memory modules, bus, storage drives, and ports. The personnel computer (PC) has a rectangular case that contains important components called hardware, many of which are integrated circuits (ICs). The fiberglass motherboard is the main printed circuit board and has a variety of important hardware mounted on it, which are connected by electrical pathways called “buses”. The CPU is the largest IC on the motherboard and contains millions of transistors. Its principal function is to execute “programs”. A Pentium^®^ 4 CPU has transistors that execute a billion instructions per second. The chipset is completely different from the CPU in design and function; it controls data and interaction of buses between the motherboard and the CPU. Memory (RAM) is fundamentally semiconductor chips storing data and instructions for access by a CPU. RAM is classified by storage capacity, access speed, data rate, and configuration.

## Computers in Radiology Practice

Computers are an integral part of modern radiology practice and are used by different radiology modalities to acquire, process, and postprocess imaging data. Computers have had a dramatic influence on contemporary radiology practice. They help in composing radiology reports, with text and images from different modalities and specialties. Their impact has extended further with the emergence of Digital Imaging and Communications in Medicine (DICOM), Picture Archiving and Communication System (PACS), Radiology information system (RIS) technology, and Teleradiology. They facilitate newer utilities like voice dictation and image reviewing on hand-held devices. For the practicing radiologist, a working knowledge of computers is an advantage. When exploited fully, they transform workflow. With this in mind, we present a basic overview of computer hardware relevant to radiology practice.

## Introduction to Computers

**Figure 1:**
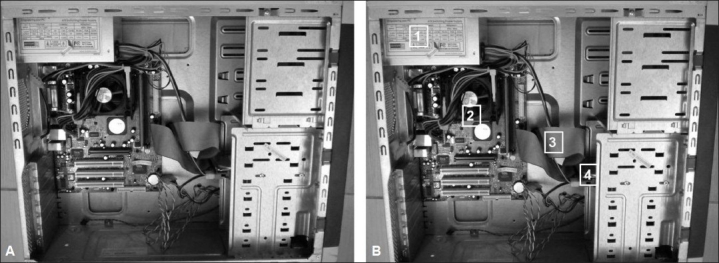
(A) Opened computer showing hardware mounted on a rectangular case. Though appearing a bit complex, the hardware items have been numerically labeled and easily identifed in B. (B) Important contents inside a computer numerically labeled: 1) SMPS Power, 2) Mother Board, 3) Cables for drives, 4) Disc Drives

The personnel computer (PC) comes in a rectangular case which contains important components, collectively called hardware [Table 1]. Different types of cases are available: desktop, slim-line desktops, mini-tower, midi-tower, full-size tower, and notebook. A type commonly seen is the midi-tower [Figure 1A and B].[1]

**Table 1:** Key hardware components in a computer[3–5]

Hardware	Functions
Motherboard	Printed circuit board with chipset, sockets, slots, and ports
CPU	Master chip for execution of program instructions, arithmetic functions, and access to memory and peripherals
Chipset	ICs controlling CPU, RAM, input/output (I/O) devices, adapter cards
RAM	Memory modules temporarily holding data and programs while the CPU processes both
Memory modules	Physical microchips holding data
Storage drives	Data-storing devices like hard and flash drives
Ports	Interface connectors for peripheral devices

Many of the hardware components are located within a computer case in the form of integrated circuits (ICs). The IC, popularly referred to as a semiconductor “chip,” is small in size and modular in design.[2] Several ICs are interconnected on a rectangular circuit board called the motherboard. Besides ICs, the motherboard also accommodates important items like power, central processor unit (CPU), memory, chipset, ports, etc. It is also interfaced with components like the keyboard, mouse, external drives, networks, etc.

For a radiologist, a basic knowledge of computer hardware components is important. Let us examine a few salient features of some important hardware.

## Motherboard

**Figure 2:**
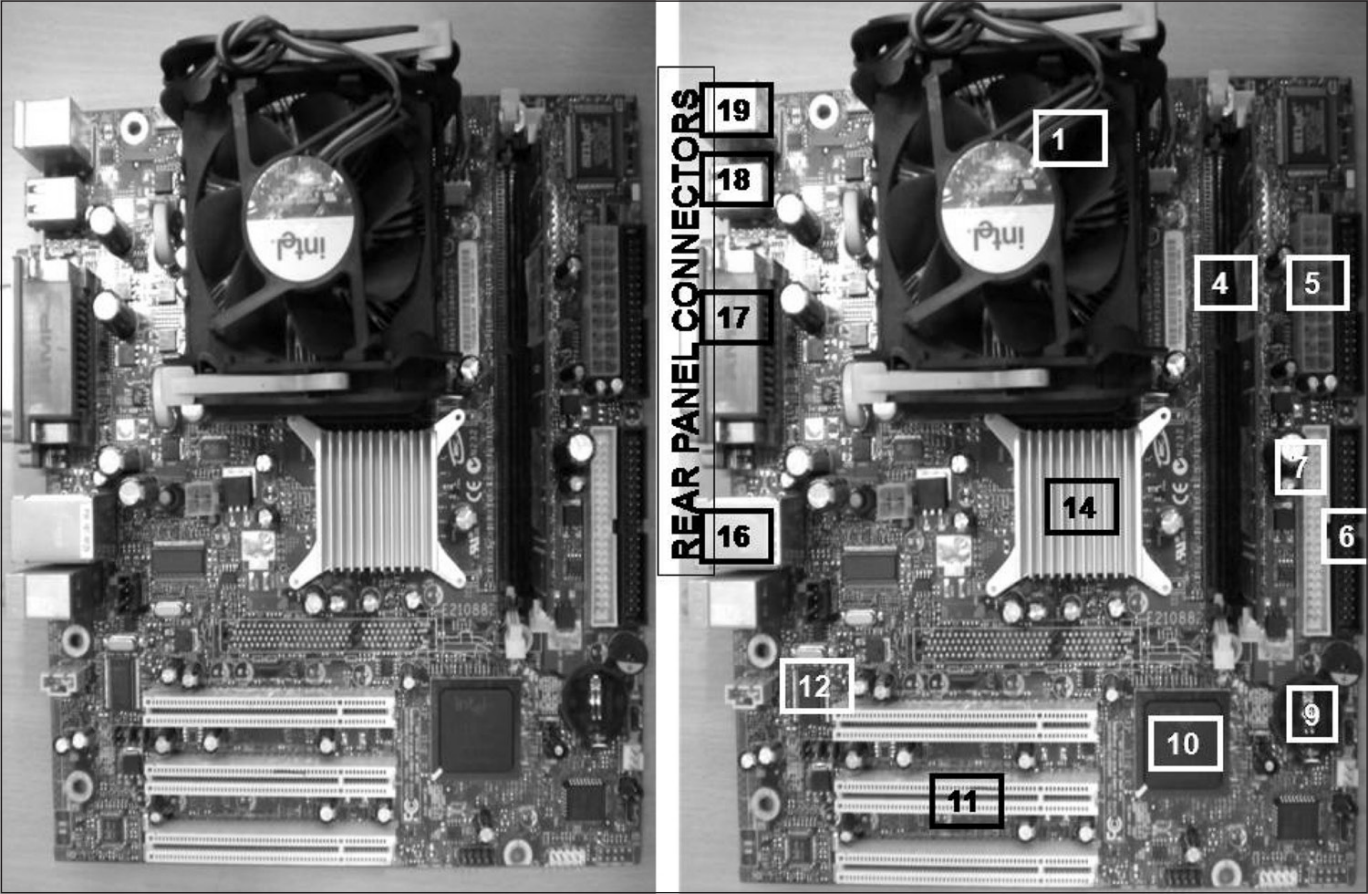
Both the panels show a motherboard. The right panel shows important components numerically labeled: 1) CPU Socket for processor, 2) Processor Core Voltage input, 3) Motherboard power input, 4) Memory Slots for memory modules, 5) Drive Connectors, 6) Primary IDE Connector (for Hard drive, CD drive upto two devices), 7) Secondary IDE Connector (for Hard drive, CD drive upto two devices), 9) CMOS Battery, 10) Chipset at Southbridge, 11) PCI Slots (for plugin of modems, network, video, sound cards), 12) BIOS chip, 13) AGP Slot for Accelerated Graphics Port card, 14) Chipset at Northbridge, 16) On-board Audio Jacks (line in, line out, microphone jacks), 17) Parallel & Serial Ports, 18) USB Ports and 19) PS2 Connectors for Keyboard & Mouse

The motherboard is the foundation of a computer. Made of fiberglass, it is the main and primary printed circuit board. It has mounted on it, important hardware components like power, CPU, memory, basic input/output system (BIOS), chipset, slots, adapter cards, ports, etc. [Figure 2].[6]

A motherboard contains many electrical pathways or “buses” which allow power and data to travel between the various hardware components by means of thin wires and cables. The electrical pathways of a bus are etched on the motherboard and connect to every part of the computer.[7] A heat sink/fan assembly is available to dissipate the heat generated.

### Issues relevant to radiology practice

A frequently used term offered to explain the size and shape of a motherboard is the “form factor.”[7] It describes the physical layout of different components and devices on a motherboard. The ATX form factor, introduced by Intel in 1995, is the most popular form factor design amongst the newer motherboards.

## Basic Output/Input System

The motherboard contains a chip called BIOS. It contains software instructions to load the basic computer hardware. It also includes a diagnostic start-up test called “power on self-test” (POST) that boots up a computer.[8] The latest PCs have the BIOS recorded on a flash memory chip – a flash BIOS.

## Complementary Metal Oxide Semiconductor

The acronym CMOS stands for complementary metal oxide semiconductor. The CMOS set-up program is part of the BIOS stored on a chip on the motherboard. It stores system information. This includes date and time, hard drive settings, boot sequence, and default settings of audio, video, ports, etc. The latest computers can automatically detect all settings.

## Central Processor Unit

Two companies, Intel and AMD, make most of the CPUs available today.[9] Though small in size, the CPU is the largest IC on a motherboard. A typical CPU has millions of transistors, all compressed together into a very small area of about 1 cm × 1 cm. Its principal function is to execute a “program.” A program is essentially “a sequence of stored instructions.”[7] Executing a program implies that “each piece of data is processed as directed by the program and the instruction set.”[7] A CPU uses a special type of memory called “cache.” When executing one step of a program, the remaining steps of instructions and related data are stored in the cache.[7]

The often quoted Moore’s law states that “the rate of the ability to add transistors to chips doubles approximately every 2 years.”[10] In 1989, a 80486 chip had 1,200,000 transistors.[11] As shown in Table 2, the growth has continued according to Moore’s law and, in 2004, a Pentium^®^ 4 chip had 125,000,000 transistors and could execute a billion instructions per second.[12] The term Celeron^®^ is given to a category of cost-effective versions of Pentium^®^ II, III, and 4 processors.[12]

**Table 2:** Generations of CPUs from Intel[12]

CPU	Year	Clock frequency (MHz)	No. of transistors
8088	1979	4.8–8	29,000
80286	1982	6–12.5	134,000
80386	1985	16–33	275,000
80486	1989	25–100	1,200,000
Pentium	1993	60–200	3,100,000
Pentium MMX	1997	166–300	4,500,000
Pentium Pro	1995	150–200	5,500,000
Pentium II	1997	233–450	7,500,000
Pentium III	1999	450–1200	28,000,000
Pentium 4	2000	1400–2200	42,000,000
	2004	2800–3600	125,000,000

Two major types of CPUs are available. They are differentiated based on the relative sizes of the instruction set processed by them. CPUs of reduced instruction set computer (RISC) type have architectures that use a small set of instructions. RISC chips are designed to execute instructions very rapidly.[7] Complex instruction set computer (CISC) is an alternative form of CPU architecture, which uses a broad set of instructions, resulting in fewer steps per operation.

An efficient CPU is the one that can quickly process a large amount of data. In practice, the efficiency of a CPU is expressed as its power. A CPUs power is determined by a) its speed and b) the amount of data it processes.[7] The speed of CPU is expressed in cycles per second since it is driven by an internal clock. Each time the clock pulses, the CPU processes an instruction. The faster the clock, the quicker a CPU processes instructions. Typically, the system clock synchronizes all activity happening on a motherboard by its continuous pulses distributed over a bus to the different components. Newer CPUs operate at speeds of megahertz (MHz or millions of cycles per second) or gigahertz (GHz or billions of cycles per second).

The amount of data a CPU processes at a time depends fundamentally on the size of the processor data bus or CPU bus.[7] A wider processor data bus makes the processor more powerful. Presently available processors have either a 32-bit or 64-bit processor data bus.

Multimedia instructions can be an in-built feature within a CPU as seen in Intel MMX processors.[7] This facilitates efficient handling of multimedia operations that normally are managed by a dedicated sound or video card.

### Issues relevant to radiology practice

Computers selected for use as consoles and workstations at radiology departments should have CPUs that routinely perform at speeds of 3 GHz or more, which means that they should be able to process over 3 billion instructions per second. This should be sufficient for efficiently handling a wide range of applications by simultaneous processing of multiple instructions.[5]

Newer processor technology advantageously incorporates more than one CPU core into a single chip. Such CPUs are described as multi-core CPUs and provide extremely high computational speed.[5] Thus, there are single-core, dual-core, quad-core processors available. Instead of a single core, each of the many cores within a CPU handles processing tasks that were earlier performed by a CPU.

More the number of cores, higher is the computational speed. As a result, each core handles processing tasks that was earlier performed by a CPU.

Such CPUs are described as multi-core CPU, which forms the basis of terms like single core, dual core, Core2Duo, quad core etc. Dual Core is a “class” or architecture of processors which refers to any processor with two physical CPU cores on the same chip. A Core2Duo is a registered trademark of Intel Corporation, representing a second generation processor offering improved efficiency for desktops and laptops.

When matched, Dual Core and Core2Duo have only one physical “technically” processor, but virtually both of them has 2 cores meaning 2 processors virtually. When compared, Core2Duo is much better than Dual Core because of a separate cache memory and with that Core2Duo offers much throughput than Dual Core. Likewise, in a QuadCore, there is one physical processor but virtually 4 cores or 4 processors.

The CPUs used in main computers at consoles of machines such as computerized tomography (CT) scanners and magnetic resonance imaging (MRIs) in radiology departments should ideally contain at least four to eight CPUs in one IC.

Notwithstanding the availability of Intel MMX, computers dealing with high-end cardiac and neurological applications at CT scan or MRI consoles or at advanced CT scan or MRI workstations should have dedicated graphic cards.

## Chipset

A chipset is an important piece of hardware, comprising a group of chips placed on the motherboard. Though completely different from CPUs in design and function, the chipset is also made of various ICs. It controls the data sent and received by a CPU. Unlike CPUs, it has other important functions like a) controlling interaction of buses between the motherboard and the CPU; b) exchanging data between the CPU and memory, hard disk drives, sound and video cards, etc; and c) determining the memory addable to a motherboard.[13]

Intel 810 is a special chipset which integrates 3D accelerated graphics and software-based audio, modem, and DVD capabilities. It offers high performance without the need for dedicated hardware cards, thereby allowing the creation of low-cost PCs.[14] Chipsets from Intel have progressively advanced, with models Intel 820, 815, 850, 845, 875P, 865, 925X, 915, 945, 955X, 965, and 975 becoming available.

### Issues relevant to radiology practice

**Figure 3:**
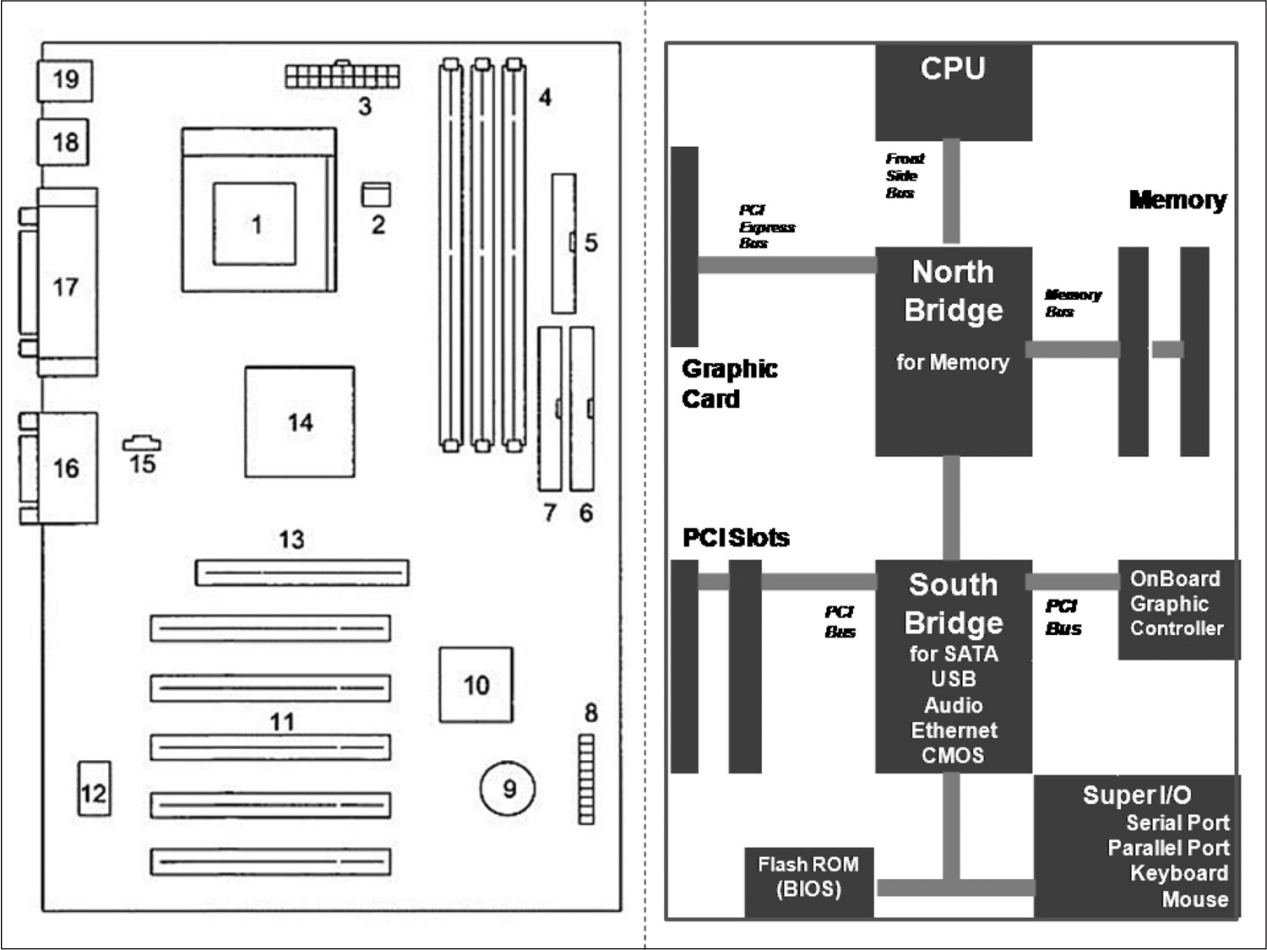
Left panel is a line diagram of a motherboard. The right panel shows a CPU, chipsets, memory, and buses. In Intel-based systems, chipsets are identified as northbridge and southbridge. In general, northbridge connections control access to memory and video card. Southbridge is an input/output device controller, handling the BIOS’ and CPU’s communication with hard drives, LAN, modem, USB ports, etc

Traditionally, the chipsets in Intel-based systems are known as the northbridge and southbridge. These two chipsets functionally perform distinct operations, while interacting with dedicated hardware components which are specifically allotted to them.[15] In general, northbridge connections control access to memory and video card, whereas southbridge is an input/output device controller and hence handles the BIOS’ and CPUs’ communication with the hard drive, LAN, modem, USB ports, etc. [Figure 3].

## Random Access Memory

Random access memory (RAM) chips are fundamentally semiconductor chips. They temporarily store data and instructions that are accessed by a CPU. Since the contents of RAM are erased when a computer is powered off, the term volatile memory is given to RAM.[7] This is in contrast to read-only memory (ROM), which permanently retains data and instructions.

**Figure 4:**
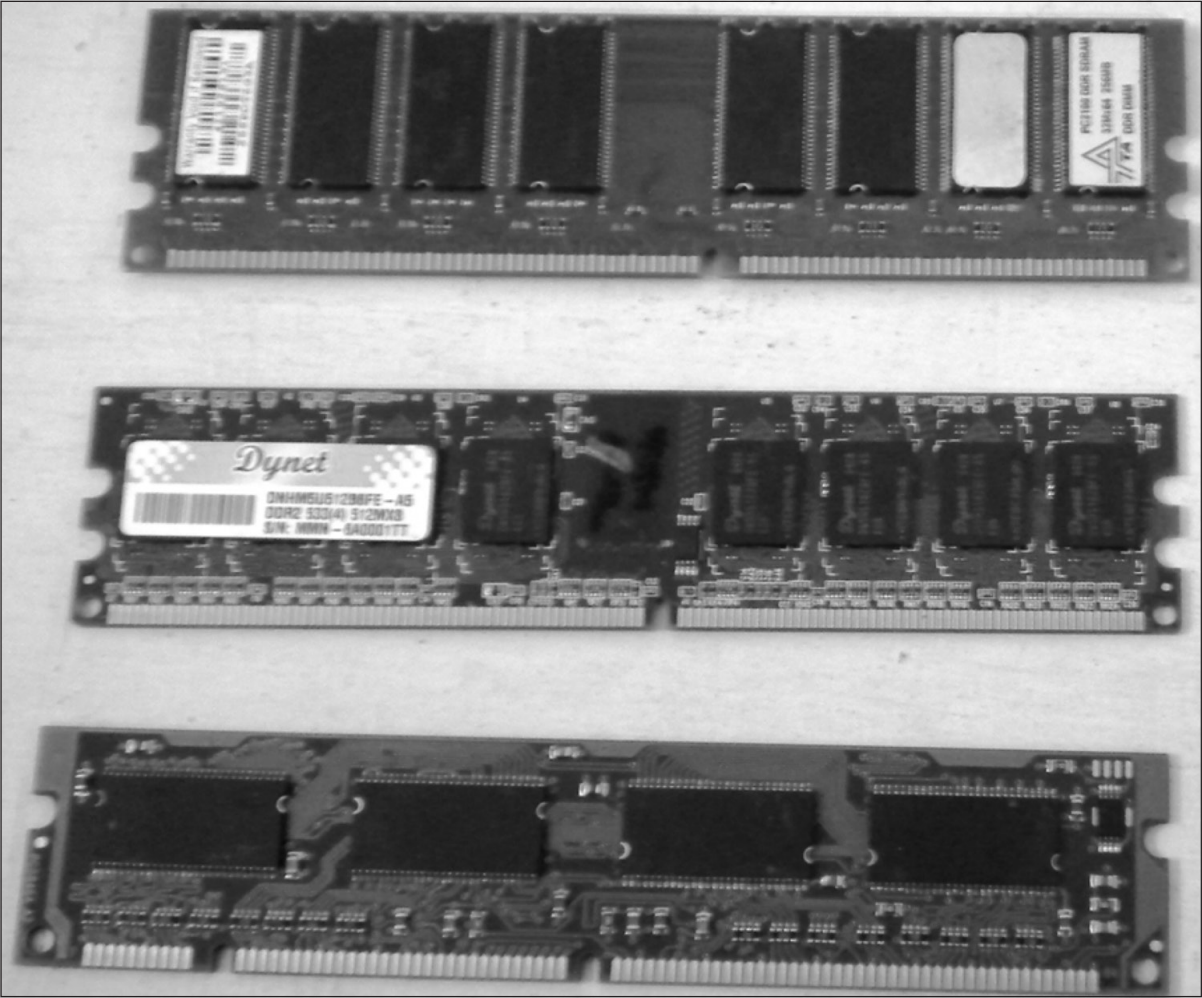
RAM chips are physical microchips located on the motherboard; they temporarily hold data and programs, while the CPU processes both. The information in RAM is lost when the PC is turned off. The displayed RAM is of DDR2 type which operates twice as fast as SDRAM

Different types of RAM are available. These are classified based on their storage capacity (in megabytes MB or gigabytes GB), access speed (in nanoseconds), data rate (DDR2) [Figure 4], and configuration [single or dual inline memory (SIMM or DIMM)].[5] Their salient features are outlined in Tables 3 and 4.

**Table 3:** Types of computer RAM based on data rate

Type of RAM	Abbreviation	Utility	Features of memory chip
Dynamic RAM	DRAM	Main memory	Needs constant refreshing
Static RAM	SRAM	Cache memory	Faster; not refreshed often
Fast page mode	FPM	Supports paging	Faster access to data
Extended data out	EDO RAM	Overlaps data accesses	Speeds up access time
Sync DRAM	SDRAM	Synchronized with memory	Data path for CPU to main memory
Double data rate	DDR SDRAM	Transfers twice per cycle	Twice as fast as SDRAM
Double data rate 2	DDR2 SDRAM	Less noise and crosstalk	Faster than DDR SDRAM memory

**Table 4:** Types of computer RAM based on configuration

Type of memory modules	Abbreviation	Features of memory chip
Dual inline package	DIP	Individual memory chip
Single inline memory module	SIMM	Circuit board with several memory chips
Dual inline memory module	DIMM	Board with SDRAM, DDR SDRAM, DDR2 SDRAM chips
RAM bus inline memory module	RIMM	Board with RDRAM chips

Memory modules are circuit boards with memory chips soldered on them. In the early 80s, computers had individual RAM chips called dual inline package (DIP) chips which were installed loosely on the motherboard. These were not only difficult to install but were fitted loosely on the motherboard and therefore frequently caused the computer to freeze or hang in mid operation.[7,16] Memory modules, by being fixed firmly on a motherboard, permanently addressed this issue.

Memory modules can be single-sided or double-sided.[17] Single-sided memory modules contain RAM on only one side of the module. Double-sided memory modules contain RAM on both sides of the module.

### Issues relevant to radiology practice

As a simple rule of thumb, “increasing the memory of a computer is the fastest, simplest, and most cost-effective method to upgrade a computer.”[18] A computer with more RAM is advantageous for improved workflow in radiology, since it has more capacity to handle different applications. The presence of more and faster RAM is useful in performing multitasking functions in real-time, as seen in typical workflow situations in radiology departments. It allows a radiographer to perform many important tasks simultaneously at a console: scanning a patient, transferring images of patients, printing films at the departmental laser printer, and burning images to a CD.

In radiology, the presence of more and faster RAM is also useful in postprocessing large 3D datasets of CT scan or MRI. It is important to remember that vendors offer more RAM at consoles, in comparison to general purpose workstations. This is the reason why a high-end dedicated workstation is to be preferred over a general-purpose workstation.

In advanced vascular, cardiac, and neurological workstations, video memory RAM (VRAM) is specifically available within video graphics cards to improve video performance.[5]

## Read-Only Memory

ROM chips retain their instructions even when the computer is shut down and their contents cannot be over-written, rewritten, or erased. ROM is a secondary storage, slower than primary, but nevertheless is a permanent form of storage. The motherboard has a few ROM chips too. These store basic instructions for starting the computer (aka booting: originating from a term in horse riding) and loading the operating system. To fulfill this function, the ROM chips contain instructions that the CPU can access directly.

## Conclusion

A working knowledge of computers is important for the radiologist as computers play an ever-increasingly important role in the “digital future” of radiology. In a nutshell, a computer offers basic functions like processing, storage of data, input and output device integration. We have dealt with processing in this article. The descriptions of other functions like storage and input and output device integration will be covered in Part II of this series.
